# Paper Biosensor for the Detection of NT-proBNP Using Silver Nanodisks as Electrochemical Labels

**DOI:** 10.3390/nano12132254

**Published:** 2022-06-30

**Authors:** Yi Peng, Nikhil Raj, Juliette W. Strasser, Richard M. Crooks

**Affiliations:** Department of Chemistry, The University of Texas at Austin, 100 E. 24th St., Stop A1590, Austin, TX 78712-1224, USA; peng.yi@wayne.edu (Y.P.); nkrjnishu01@gmail.com (N.R.); jstrasser@utexas.edu (J.W.S.)

**Keywords:** silver nanodisks, NT-proBNP, metalloimmunoassay, electrochemical label, galvanic exchange

## Abstract

We report on the use of silver nanodisks (AgNDs), having a diameter of 50 ± 8 nm and a thickness of 8 ± 2 nm, as electrochemical labels for the detection of a model metalloimmunoassay for the heart failure biomarker NT-proBNP. The detection method is based on an electrochemically activated galvanic exchange (GE) followed by the detection of Ag using anodic stripping voltammetry (ASV). The AgNDs labels are superior to Ag nanocubes and Ag nanospheres in terms of the dynamic range for both the model and NT-proBNP metalloimmunoassays. The linear dynamic range for the model composite is 1.5 to 30.0 pM AgNDs. When AgND labels are used for the NT-proBNP assay, the dynamic range is 0.03–4.0 nM NT-proBNP. The latter range fully overlaps the risk stratification range for heart failure from 53 pM to 590 pM. The performance improvement of the AgNDs is a result of the specific GE mechanism for nanodisks. Specifically, GE is complete across the face of the AgNDs, leaving behind an incompletely exchanged ring structure composed of both Ag and Au.

## 1. Introduction

For several years our group has been developing a home-use sensor for the heart failure biomarker NT-proBNP. NT-proBNP is a cardiac peptide hormone produced by the ventricular myocardium during volume overload [1,2,3,4,5,6]. The purpose of the sensor is to allow individuals diagnosed with Stage 2 or 3 heart failure [7] to quantitatively monitor the status of their disease at home. There are many difficulties associated with the development of quantitative home-use sensors, particularly when the biomarker is present in the blood at very low concentrations [8,9]. For example, the required dynamic range for NT-proBNP risk stratification is 53 pM (450 pg/mL) to 590 pM (5000 pg/mL) [6], and a concentration of 116 pM (1000 pg/mL) is used as a baseline for diagnosis and therapy [3,10]. In addition to the rather low limit of detection (LOD) required for NT-proBNP, other obstacles for home-use sensors that must be overcome are cost, the time required for the bioassay, robustness of the assay, and the ease of use.

**Scheme 1:**
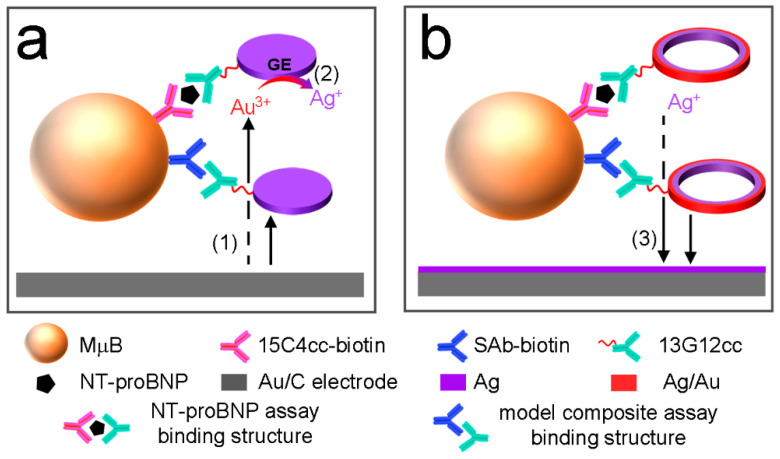
Illustration of the electrochemically induced GE mechanism used for assay detection. (**a**) Electrochemically activated GE and (**b**) the following Ag electrodeposition process.

Although several methods have been reported for detection of NT-proBNP, including ELISA [11], fluorescence [12], and electrochemiluminescence [13], they are not as adaptable to home use as electrochemical methods (which satisfy the need for rapid, specific, inexpensive, and automated means of detection) [8,14,15,16,17]. Up to this point, we have been able to address most of the foregoing obstacles [18,19,20,21,22], but achieving the necessary LOD for NT-proBNP has been a challenge. The sensor itself is electrochemical and relies on a metalloimmunoassay. As shown in Scheme 1, the bioassay consists of three parts: a capture antibody (Ab) conjugated to a magnetic microbead (MµB), the target (NT-proBNP), and a signaling Ab conjugated to a Ag nanoparticle (NP) label. In operation, the immuno-sandwich is enriched on an Au-coated electrode via a magnetic force. Next, the Au coating is electrochemically oxidized to Au^3+^, which diffuses into the vicinity of the AgNPs. There, it undergoes galvanic exchange (GE, Au^3+^ + 3 Ag → Au + 3 Ag^+^) with the AgNPs due to the higher redox potential of Au/Au^3+^ vs. Ag/Ag^+^ [23]. The resulting Ag^+^ is then electrochemically reduced and concentrated onto the electrode surface. Finally, the electrode-confined Ag is oxidized electrochemically and the resulting charge, which is related to the amount of NT-proBNP present, is determined.

In our initial studies we used 20 nm spherical AgNPs as the electroactive labels, but for a number of reasons, it was not possible to achieve the necessary LOD of 116 pM [20]. The most fundamental reason for this is related to the GE process itself. Specifically, during GE, we have found that a skin of Au deposits over the AgNP, thereby decreasing the total number of equivalents of available Ag [18,24]. We recently addressed this problem by using two different types of Ag labels simultaneously: spherical AgNPs and Ag nanocubes [25]. This approach was effective, because the Ag nanocubes are sensitive to the lower concentrations of NT-proBNP, while the spherical AgNPs are effective at the higher end of the risk stratification range [26]. The more general conclusion of this study was that the dynamic range of metalloimmunoassay can be tuned simply by changing the shape of the metallic labels.

While the foregoing approach was effective, we thought that it might be possible to design a single-shaped AgNP label that would cover the entire risk stratification range for NT-proBNP. Considering that the problem with the Ag spheres involved the formation of an Au skin that prevented full GE, we hypothesized that a shape having a large surface area with minimal interior Ag atoms would be the most effective label. Accordingly, we synthesized and tested Ag nanodisk (AgND) labels and found that they are highly effective and lead to full coverage of the required range for NT-proBNP.

## 2. Materials and Methods

### 2.1. Chemicals and Materials

NaOH, HCl, NaIO_4_, Na_2_HPO_4_, NaH_2_PO_4_·H_2_O, sodium citrate, Whatman grade 1 chromatography paper (180 µm thick, 20 cm × 20 cm sheets, a linear flow rate of water = 0.43 cm/min), and siliconized low-retention microcentrifuge tubes were purchased from Fisher Scientific (Pittsburgh, PA, USA). Ascorbic acid was purchased from ACROS (Morris Plains, NJ, USA). Polyvinylpyrrolidone K30 (PVP) was purchased from MP Biomedicals (Solon, OH, USA). HAuCl_4_, AgNO_3_, H_2_O_2_, NaBH_4_, glycerol, phosphate-buffered saline (PBS, pH = 7.4), and bovine serum albumin (BSA) were purchased from Sigma Aldrich (St Louis, MO, USA). Human serum, unfiltered (hereafter, just “serum”), was from Millipore-Sigma (Taunton, MA, USA).

Conductive carbon paste (Cl-2042) came from Engineered Conductive Materials (Delaware, OH, USA). 1.0 µm-diameter magnetic beads, streptavidin-coated, (MµBs, Dynabeads, MyOne Streptavidin T1, 10 mg/mL) were purchased from from Invitrogen (Grand Island, NY, USA). NT-proBNP, N-terminal prohormone brain natriuretic peptide, monoclonal immunoglobulin G anti-NT-proBNP 13G12cc, and the 15C4cc capture Ab were purchased from HyTest (Turku, Finland). Abcam (Cambridge, UK) provided the biotinylated polyclonal anti-mouse immunoglobulin G secondary antibody (SAb). Deionized (DI) water (>18.0 MΩ-cm, Milli-Q Gradient System, Millipore, Burlington, MA) was used for all solutions. The PBS concentration was 1× unless noted otherwise (10 mM phosphate buffer plus 2.7 mM KCl and 137 mM NaCl).

### 2.2. Instrumentation

UV-vis measurements were carried out using a Hewlett-Packard HP8453 spectrometer having a micro-quartz cuvette (50 µL, Hellma, Müllheim, Germany). Incubation steps for bioconjugation involved a Thermo Scientific tube revolver and a BioShake iQ (Quantifoil Instruments, GmbH, Jena, Germany). Neodymium magnets were used for washing and separating steps involving magnetic microbeads (MµBs) (purchased from K&J Magnetics, Pipersville, PA, USA).

### 2.3. Synthesis of Silver Nanodisks (AgNDs)

AgNDs were synthesized using a seed-mediated method. First, Ag seeds were synthesized by adapting a published method [27]. Briefly, 100 µL AgNO_3_ (50.0 mM), 1.0 mL sodium citrate (75.0 mM), 200 µL glycerol (17.5 mM), and 120 µL H_2_O_2_ (35%) were added into 48.0 mL H_2_O with magnetic stirring. After 1.0 min, 0.5 mL NaBH_4_ (100 mM) was added into the above solution to reduce Ag^+^ to form Ag seeds (Figure S1). The solution was stirred in air overnight to remove unreacted NaBH_4_. 

The seed-mediated growth step was carried out as follows. First, 43.0 mL H_2_O and 1.0 mL sodium citrate (75.0 mM) were added to the seed solution. Second, 3.0 mL AgNO_3_ (8.33 mM) and 3.0 mL ascorbic acid (33.3 mM) were added at a rate of 100 µL/min. Third, the resulting solution was stirred for another 30 min to ensure complete growth of the AgNDs. To exclude unreacted reagents, the products were filtered using 10 kDa molecular weight cut-off spin filters. The filtered AgNDs were dispersed in 1.0 mM sodium citrate. The Ag concentration was measured by inductively coupled plasma mass spectrometry (ICP−MS, 7500 ce, Agilent, Santa Clara, CA, USA).

### 2.4. Preparation of Assay Components

Before the 13G12cc Ab was bioconjugated onto the AgNDs, a heterobifunctional cross-linker (HBCL), which was synthesized by a previously published protocol [19], was used to passivate the AgND surface. Briefly, 25.0 µL HBCL (5.0 mM) was added to 500 µL AgNDs and incubated for 20 min at 600 rpm at room temperature (RT, 22 ± 3 °C). Next, 20.0 µL HBCL-modified 13G12cc Ab (1.33 µM, the protocol for bioconjugating 13G12cc Ab onto HBCL can be found in the Supplementary Materials) was added and incubated for 30 min, followed by back-filling with 50.0 µL polyvinylpyrrolidone (PVP, 10 mg/mL) for 20 min. Excess conjugation reagents were then removed by centrifugation for 20 min at 13,000× *g* at 4 °C. The formed conjugate was redispersed in 500 µL of 1.0 mM sodium citrate. Henceforth, this will be referred to as the AgND−13G12cc conjugate. 

For the model assay, the biotinylated SAb was conjugated to streptavidin-coated MµBs using the protocol provided by the manufacturer [28]. Specifically, 100 µL of MµBs (~7−10 × 10^9^ MµBs/mL) were washed with 0.01 M pH = 7.4 phosphate buffer without NaCl (hereafter just “PB”) by magnetic separation and then redispersed in PB. Next, 40.0 µL of 6.67 µM SAb was added and incubated for 30 min at 30 rpm at RT using a tube revolver. Finally, the conjugated MµBs were washed by magnetic separation five times with 100 µL PB containing 1% *w*/*v* BSA and redispersed in a final volume of 100 µL PB containing 1% *w*/*v* BSA. The resulting product is referred to as MµB-SAb.

The 15C4cc capture Ab for the NT-proBNP assay was biotinylated using the protocol and a kit provided by the vendor [29]. Next, the modified Ab and the streptavidin-coated MµBs were conjugated using the procedure mentioned earlier for MµB-SAb. The final conjugate is referred to as MµB-15C4cc.

### 2.5. Formation of the Metalloimmunoassay

Two different metalloimmunoassays were synthesized: the model assay and the antigen-specific assay. The model assay was prepared by bioconjugating MµB-SAb and AgNP-Ab via attached Abs. Specifically, 16.0 µL of MµB-SAb were combined with 100 µL of AgNP-Ab in 1.0 mM sodium citrate or serum. The conjugate was then incubated in a tube revolver (30 rpm) for 30 min. 1.0 mM sodium citrate was used to wash the product five times (via magnetic separation). The conjugate was then dispersed in 16.0 µL of 1.0 mM sodium citrate. Henceforth, this conjugate will be denoted as the MµB-AgNP model composite (MµB-AgNP MC).

The antigen-specific assay was prepared step-wise. This assay was prepared in a microcentrifuge tube, blocked with SBB, as follows. First, the MµB−15C4cc conjugate (16.0 µL) was pipetted into a tube with 100 µL NT-proBNP, present at a known concentration, in PBS or undiluted normal serum. These components were incubated at 30 rpm at RT for 30 min. The partially formed assay was washed by magnetic separation three times with 1% (*v*/*v*) Tween-20/PBS to eliminate unbound NT-proBNP. Finally, the AgNP−Ab conjugate (100 µL) was added. This mixture was incubated at 30 rpm for 30 min and then washed. The fully formed antigen-specific assay was then suspended in a PBS volume of 16.0 µL.

### 2.6. Electrochemistry

Prior to analysis, 2.0 µL aliquots of either the model assay or the antigen-specific assay were suspended in 48 µL of 1× PBS (pH = 7.4) to provide a sample volume of 50.0 µL. The diluted samples were then conveyed to the sensor platform, the formed assays were separated onto the WE for ~30 s using the magnet force, and finally electrochemical assay was carried out as follows. 

A CH Instruments model 760B electrochemical workstation (Austin, TX, USA) was used for electrochemical measurements. The ESI discusses the preparation of the paper electrodes and also the loading of AuNPs onto the WE. These processes have previously been described [25]. The electrochemical measurements were similar to those used in previous studies (but with some optimization) [18,20,21]. To initiate GE, the WE potential was moved from 0 to 0.80 V for 12.0 s (which oxidizes Au^0^ to Au^3+^). Next, the potential was changed from 0 to −0.70 V for 100 s (Figure S2a) to reduce Ag^+^ to Ag^0^. These steps were repeated three times (Figure S2b), and then the electrode potential was scanned twice from −0.70 to 0.20 V at 50.0 mV/s to oxidize Ag^0^. The charge arising from the second ASV scan was determined by integration, and it corresponds to the output signal of the sensor. The electrochemical measurements were performed using 1× PBS (pH = 7.4). The potentials are vs. a carbon quasi-reference electrode (CQRE).

## 3. Results

### 3.1. Characterization of AgNDs

**Figure 1:**
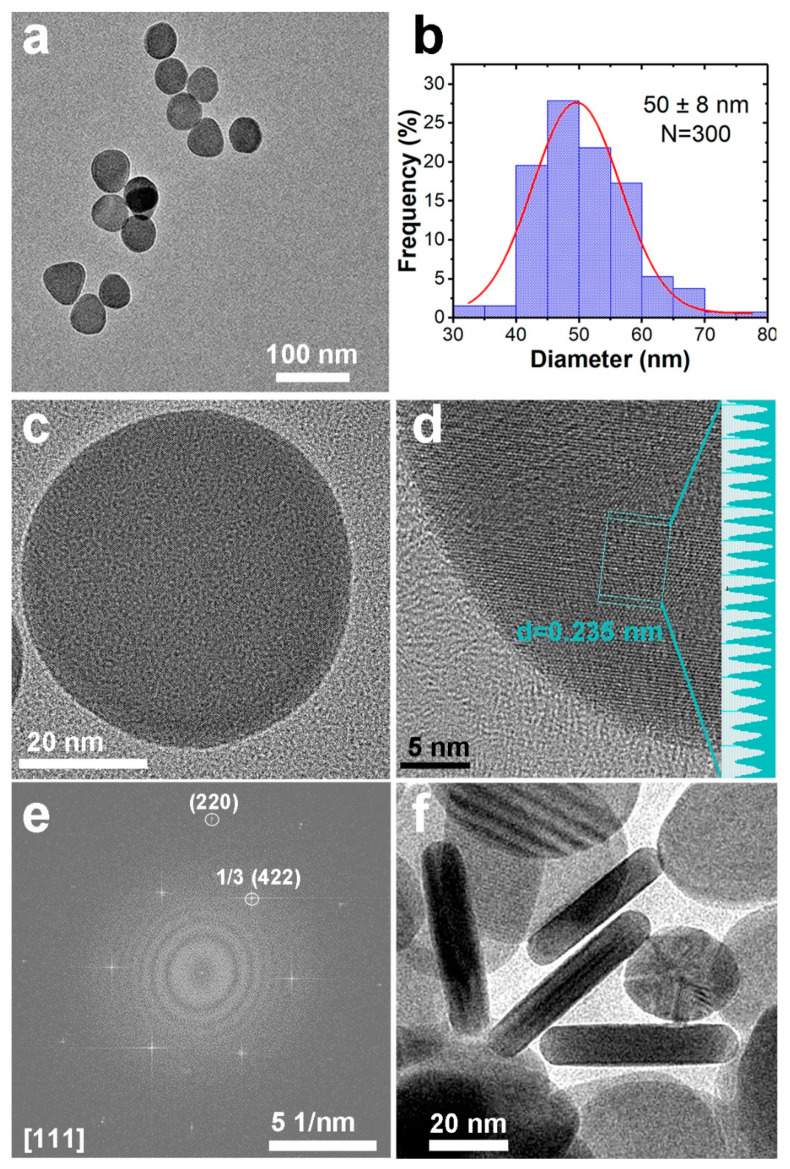
Characterization of the AgND labels. (**a**) Representative TEM image of the as-prepared AgNDs. (**b**) Histogram of the AgND diameter obtained by measuring 300 randomly selected AgND particles. (**c**) An expanded TEM image of a single AgND. (**d**) HRTEM image of part of the AgND face. (**e**) The corresponding selected area electron diffraction (SAED) pattern via fast Fourier transform of the particle shown in frame c. (**f**) A typical TEM image showing some AgNDs oriented perpendicular to the grid.

The seed-mediated synthetic method for preparing AgNDs is provided in the Experimental Section. Figure 1a is a TEM image of the resulting materials, and it reveals that most of the individual AgNDs are round, but a few have a polygonal shape. Figure 1b is a histogram, obtained by measuring the diameters of 300 randomly selected particles, showing that the diameter of the AgNDs is 50 ± 8 nm. Figure 1c is an image of a single particle, and it shows that the electron contrast is nearly uniform across the surface of the disk. This suggests that the disk thickness is also nearly uniform. A high-resolution TEM (HRTEM) image of part of a AgND face is shown in Figure 1d, and it indicates a lattice spacing of 0.235 nm, which is assigned to the (111) facet of fcc Ag (PDF card #4-783) [30].

The selected area electron diffraction (SAED) pattern of an AgND along the [111] projection is shown in Figure 1e. It exhibits the 1/3(422) and (220) spots of the Ag fcc crystalline structure [31]. As shown in Figure 1f, we were sometimes able to find AgNDs that were situated perpendicular to the TEM grid. The rod-like projection further confirms the disk morphology of the AgNDs. From images such as that shown in Figure 1f, the thickness of the disks is found to be 8 ± 2 nm (based on 20 AgNDs). Figure S3 provides the HRTEM of the AgND narrow edge and the corresponding SAED pattern, which exhibits the characteristic (111) spot of the Ag fcc crystalline structure. This additional analysis confirms the fcc crystal phase. 

### 3.2. Bioconjugation of Antibodies to AgNDs

Before conjugating Abs to the AgNDs, the concentration of AgNDs was determined. This was done by measuring the total number of Ag atoms in a particular solution volume by ICP-MS and then dividing this value by the average number of Ag atoms in an individual AgND (9.2 × 10^5^, estimated from the average geometrical dimensions of an individual AgND). The resulting concentration of the stock solution of AgNDs is 230 pM.

**Figure 2:**
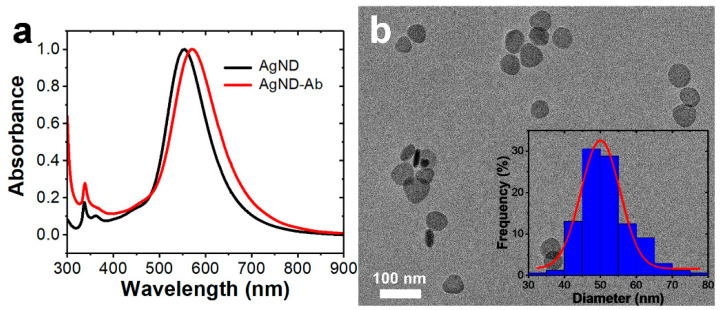
(**a**) UV−vis spectra of solutions of as-prepared AgNDs and AgND-Ab conjugates. All spectra were obtained in 1.0 mM sodium citrate solution using a 50.0 μL cuvette having a 1.00 cm pathlength. (**b**) Representative TEM image showing the AgNDs after modification with Abs (AgND-Abs). The inset is a histogram of AgND diameter obtained by measuring 200 randomly selected AgND-Ab particles. The AgND-Abs were prepared in the presence of PVP but in the absence of Cl^−^. They were dispersed in a 10.0 mM sodium citrate solution.

A UV-vis spectrum of a diluted AgND solution (black) is shown in Figure 2a. It exhibits a major peak around 553 nm arising from the in-plane dipole plasmon resonance mode. A second minor peak at ~338 nm, which has a small shoulder at ~362 nm, is attributable to the out-plane quadrupole and dipole plasmon resonance modes [32]. After bioconjugating Abs onto the AgNDs (the conjugate is denoted as AgND-Ab), the major peak in the UV-vis spectrum (red, Figure 2a) shifts from 553 to 569 nm, but its absorbance does not change. The 16 nm peak shift is a characteristic of bioconjugated AgNDs, and it arises from changes to the refractive index of the environment surrounding the AgND [33]. This blue shift of the SPR peak has also been observed in the cases of spherical AgNPs and Ag nanocubes [19,25]. Because the absorbance of the major peak does not change, it is possible to use it to determine the concentration of AgNDs following conjugation. Accordingly, a calibration curve was created from UV-vis spectra (like that shown in Figure 2a) for a series of dilute AgND solutions (Figure S4).

During bioconjugation, PVP was used as a back-filling reagent to stabilize the AgNDs. Figure S5 compares the UV-vis spectra of the AgND-Ab conjugate in the presence and absence of PVP in PB. The key point is that the major absorbance peak broadens and its intensity attenuates if PVP is not present. These spectral changes confirm the importance of PVP for stabilizing the AgND morphology. Another important point is that Cl^−^ can also affect the stability of the AgND-Ab conjugate. As shown in Figure S6a, the intensity of the main absorbance peak decreases and shifts to lower wavelengths as a function of time when the AgND-Ab conjugate (backfilled with PVP) is stored in PBS (which contains NaCl). These changes indicate a significant morphological instability for times exceeding 1 h. In contrast, when AgND-Ab is stored in a Cl^−^-free buffer, the UV-vis spectra exhibit almost no change after two weeks (Figure S6b). Accordingly, we used PB rather than PBS in the remainder of this study. The key point is that we observed no change to the size or morphology of the AgND-Ab conjugate when they are stabilized with PVP and kept free of Cl^−^ (Figure 2b).

### 3.3. Formation of Model Composites

Before moving on to study the NT-proBNP assay, we examined a simpler model composite. It consists of Ab-functionalized MµBs directly conjugated to Ab-functionalized AgNDs, and it is hereafter denoted as the MµB-AgNP MC. As discussed next, the formation of the MµB-AgNP MC was confirmed by both UV-vis spectroscopy and electrochemical measurements. 

**Figure 3:**
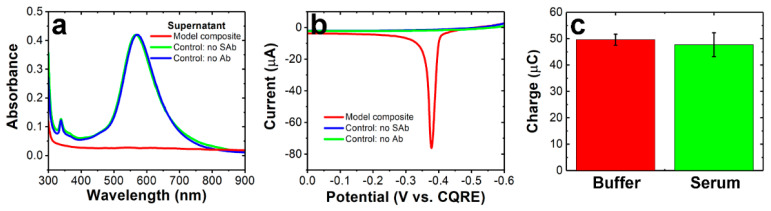
Demonstration of formation of the model composite using a AgND concentration of 25 pM. (**a**) UV-vis spectra of supernatants of solutions obtained after magnetic separation of the conjugate. The two control experiments are performed in the absence of SAbs on the MµBs or without Abs on AgNDs. All spectra were obtained in 1.0 mM sodium citrate solution using a 50.0 µL cuvette having a 1.00 cm pathlength. (**b**) Representative ASVs of the corresponding model composite were collected by magnetic separation. (**c**) Comparison of charges measured using the model assay formed in buffer and human serum. The error bars represent the standard deviation from the mean for three independent measurements.

After forming the MµB-AgNP MC assay and collecting it with a magnet, the UV-vis spectrum of the supernatant was obtained. As shown by the red spectrum in Figure 3a, no absorption features were observed, indicating complete conjugation of the AgNDs to the MµBs. In contrast, when the same experiment is performed, but in the absence of the SAb on the MμB, the spectrum of the supernatant (green, Figure 3a) after separation of the MμBs reveals a strong absorbance corresponding to the presence of AgNDs. The comparison of the absorbance of the peak in Figure 3a to the calibration curve (Figure S4) indicates that the AgND concentration in the supernatant is ~25 pM. In other words, in the absence of an Ab binding partner on the MµBs, all the AgNDs remain in the supernatant. A similar control experiment was carried out by excluding the Abs on the AgNDs, and the result was the same (blue spectrum, Figure 3a). On the basis of these UV-vis experiments, we conclude that there is little or no nonspecific adsorption of the AgNDs to the other components of the MµB-AgNP MC and that the Abs used for the MµB-AgNP MC effectively conjugate the AgNDs to the MµBs.

The general procedure for carrying out electrochemical experiments using the MµB-AgNP MC is provided in the Experimental Section. More specifically, however, the same three experiments, discussed in the previous paragraph in the context of UV-vis spectroscopy, were carried out using electrochemistry. The results are shown in Figure 3b. In this case, the 25 pM MµB-AgNP MC leads to a prominent ASV peak corresponding to the oxidation of the AgNDs. The two control experiments, however, exhibit no detectable faradaic current. These electrochemical results are fully consistent with the UV-vis data, indicating conjugation of the MµB-AgNP MC only in the presence of the two Abs.

The foregoing experiments were carried out in buffer, but we also prepared the MµB-AgNP MC in human serum and compared the electrochemical results to those obtained in buffer. Following the formation of the MµB-AgNP MC in serum, the samples were collected by magnetic separation and washed with PB containing 1% BSA. As shown in Figure 3c, the charges obtained by integrating the ASV peaks for the MµB-AgNP MC prepared in buffer or serum are almost identical. This finding indicates the MµB-AgNP MC can be effectively detected in human serum (but washing is required prior to electrochemical analysis).

### 3.4. Detection of Model Composites

**Figure 4:**
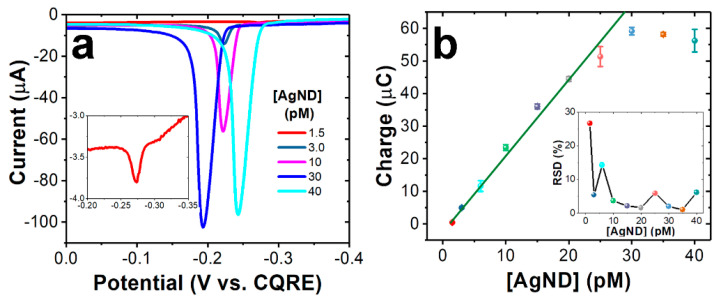
Detection of different concentrations of the model composites. (**a**) Representative ASVs for model composites having the concentrations indicated in the legend. The inset is an expanded view of the ASV of a sample with 1.5 pM AgNDs. (**b**) A calibration curve for the model composite. The linear range is 1.5–30.0 pM. The error bars represent the standard deviation from the mean for three independent measurements. The corresponding relative standard deviations as a function of the model composite concentration are shown inset.

After confirming formation of the model composite, we examined the effectiveness of the AgNDs for detection. This study was carried out by preparing a series of AgND-Ab solutions having different concentrations and then measuring the electrochemical response using the paper sensing device. Figure 4a shows representative ASVs of MµB-AgNP MCs having concentrations of AgNDs ranging from 1.5 pM to 40 pM. Qualitatively, the Ag oxidation peaks have two notable features. First, the peak currents increase with increasing concentration of AgNDs, and second, the peak potentials shift randomly. The latter observation is a consequence of the somewhat variable CQRE used to set the reference potential. The lowest detectable concentration of AgNDs is 1.5 pM, as shown in the inset of Figure 4a.

Figure 4b is a plot of the integrated current (i.e., charge) of ASVs like those shown in Figure 4a as a function of the concentration of the AgNDs. In the concentration range of 1.5 pM to 30.0 pM, the plot is linear with an R^2^ value of 0.98. The inset of Figure 4b shows that the relative standard deviation (RSD) of the average charge values is <10%, except for two of the three lowest concentrations. The findings in Figure 4 encouraged us to examine next the full range of NT-proBNP concentrations relevant to clinical applications.

### 3.5. Detection of NT-proBNP

The primary objective of this study is to achieve detection of NT-proBNP between 53 pM and 590 pM, which is the range required for risk stratification [6]. The type of antibodies used for this assay and the precise assay procedure are provided in the Experimental Section. 

**Figure 5:**
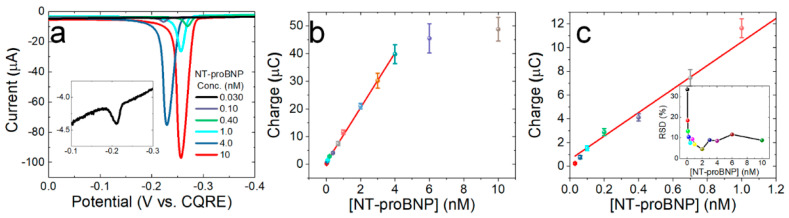
Detection of different concentrations of NT-proBNP using the antigen-specific assay. (**a**) Selected ASVs for the antigen-specific composites formed using the indicated NT-proBNP concentrations. The inset is an expanded view of the ASV for the 30.0 pM NT-proBNP sample. (**b**) Calibration curve covering the entire range tested for NT-proBNP. (**c**) Expanded view showing more clinically relevant range (0.03–1.0 nM) of the calibration curve in (**b**). The error bars represent the standard deviation from the mean for three independent measurements. The corresponding relative standard deviations as a function of NT-proBNP concentration are shown inset.

Figure 5a shows representative ASVs for composites obtained using NT-proBNP concentrations ranging from 30.0 pM to 10.0 nM, which more than covers the risk stratification range. The ASV for the lowest detectable concentration of 30.0 pM is shown in Figure 5a inset. Figure 5b is a calibration curve for the charge, obtained by integrating ASV peaks like those in Figure 5a, vs. the concentration of NT-proBNP. The plot is linear over the NT-proBNP concentration range of 30.0 pM to 4.0 nM (R^2^ = 0.99). At concentrations > 4.0 nM, however, the charge signal plateaus. Figure 5c is an expanded view of the calibration curve at the lower NT-proBNP concentrations. The RSDs for the points on the linear parts of the calibration curves (Figure 5c inset) are <20% except for 30.0 pM, which is below the lower limit for risk stratification.

### 3.6. GE Mechanism for AgNDs

To better understand how the AgND labels affect the metalloimmunoassay, we calculated the Ag collection efficiency (AgCE%) for the MµB-AgNP MC assay using the data shown in Figure 4 [25]. The AgCE% is defined as the percentage of Ag measured (recovered) by ASV divided by the total amount of Ag present in the sample. The AgCE% is an important factor because it can affect the dynamic range of the sensor. For instance, a dynamic range of 12 pM to 498 pM of Ag labels was obtained using 20 nm spherical AgNPs for the MµB-AgNP MC assay. In that case, the AgCE% was ~16% [18,21]. Using Ag nanocubes as labels, rather than spheres, for the MµB-AgNP MC assay narrowed the dynamic range to 0.4–4.7 pM of Ag labels due to the increased AgCE% (~57%) [25].

**Figure 6:**
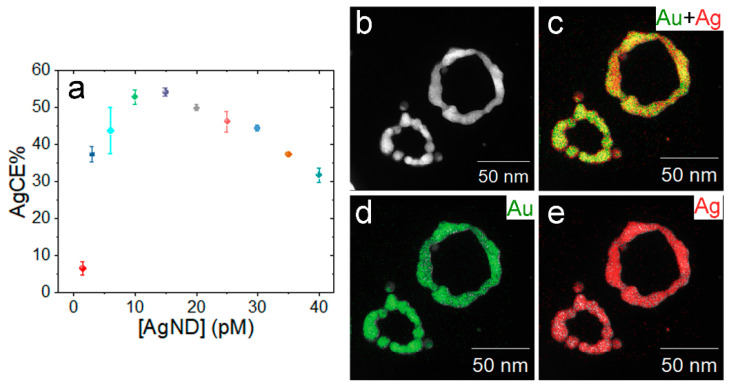
Confirmation of the GE mechanism for the MµB-AgNP MC assay. (**a**) AgCE% vs. the concentration of the AgNDs derived from the results shown in Figure 4. (**b**) Typical dark-field STEM image of the AgNDs following GE. As illustrated in Scheme 1, the AgNDs are converted to ring structures following GE. (**c**–**e**) EDS maps of ring structures shown in (**b**) for the elements indicated in the legends. Energy dispersive spectroscopy (EDS) was performed using a JEOL NEOARM TEM at an accelerating voltage of 80 kV with a point-to-point STEM resolution of 0.11 nm.

Figure 6a is a plot of AgCE% as a function of AgND concentration for the MµB-AgNP MC assay. Most of the data points in Figure 6a have an AgCE% higher than 30%, and the maximum AgCE% is 54% when the AgND concentration is 15.0 pM. The latter value for the AgCE% is higher than we measured for spherical AgNPs (~16%) [18,24] and similar to that of Ag nanocubes (~57%) [25].

We have already established that the AgCE% is dependent on the morphology of the Ag labels. This is a consequence of the means by which GE takes place on the different shapes. To better understand how GE takes place on AgNDs, we characterized AgNDs following GE using STEM and EDS. Figure S7 and Figure 6b–e show that following GE the AgNDs convert into nanorings composed of Ag and Au. The average compositions are 78% Au and 22% Ag with standard deviations of 9% calculated using 12 randomly selected nanorings.

Conversion of the disk shape to a ring (e.g., dissolution of the center of the disk) is consistent with efficient GE and the electrochemical results discussed earlier. We attribute the improvement in AgCE% primarily to the nanodisk shape. Briefly, the flat faces of AgNDs have (111) facets, which have smaller free energy compared to the narrow edges with (110) facets exposed [34]. Therefore, the stabilizing citrate capping ligands bind more strongly on the (110) facets. This results in stabilization of the edges of the disk, but leaves the (111) facets on the faces of the disks more vulnerable to GE. This same mechanism has been invoked to account for the formation of triangular nanorings upon GE of triangular Ag nanoprisms [31,35,36,37].

## 4. Summary and Conclusions

In summary, we have investigated the sensing performance of ~50 nm AgND electrochemical labels for metalloimmunoassay for both a model composite (MµB-AgNP MC) and NT-proBNP. Due to the nature of the GE of Au^3+^ and Ag, the AgNDs provide a dynamic range of 1.5 pM to 30.0 pM for the MµB-AgNP MC. In previous reports, we have used Ag nanocube and Ag sphere labels. The former provided a low limit of detection, but a modest dynamic range of 0.4 pM to 4.7 pM [25]. The latter provided a wide dynamic range, but a rather high limit of detection: 12 pM to 498 pM [21]. Neither of these ranges were appropriate for our ultimate goal of detecting NT-proBNP in the risk stratification range.

The results discussed in the previous paragraph encouraged us to try using AgNDs for NT-proBNP assay. Indeed, we found a linear dynamic range of 30.0 pM to 4.0 nM, which more than covers the risk stratification range for clinical applications of 53 pM to 590 pM. Moreover, the sensitivity of the calibration curve (Figure 5c), which is 2.1 μC/pM of NT-proBNP, is rather good. We attribute these improvements to the enhanced GE efficiency of the AgNDs compared to either the nanospheres or nanocubes discussed in previous reports [20,25]. The evidence for nearly complete GE is clearly demonstrated by the conversion of the disks to hollow rings (Figure 6).

## Data Availability

The data presented in this study are available on request from the corresponding authors.

## Supplementary Materials

The following supporting information can be downloaded at: https://www.mdpi.com/article/10.3390/nano12132254/s1, Additional experimental information; Figure S1. Representative TEM image of the Ag seeds used to prepare the AgNDs. Figure S2. Optimization of the Ag electrodeposition time and number of GE cycles for the MµB-AgND MC assay. The experiment was carried out using 10 pM AgNDs and different (a) electrodeposition times and (b) number of GE cycles prior to the ASV measurements. Figure S3. (a) HRTEM image of part of the AgND narrow edge. (b) The corresponding selected area electron diffraction (SAED) pattern via fast Fourier transform of the particle shown in (a). Figure S4. Calibration curve established using the absorption intensity of the major peak at 553 nm and the concentration of AgNDs obtained from ICP-MS. (a) The UV-vis spectra of AgND solutions having the concentrations indicated in the legend. All spectra were collected using a 50.0 μL cuvette having a 1.00 cm pathlength. (b) The corresponding linear plot with a linear function of *y* = 0.0165*x* + 0.0071, where y is the absorption intensity and *x* is the concentration of AgND with a unit of pM. Figure S5. Effect of PVP on AgND stability. The UV-vis spectra of the AgND-Ab conjugate (~27 pM) in the presence (black curve) and absence (red curve) of PVP. The spectra were collected in phosphate buffer solutions (no Cl^−^ present). All spectra were collected using a 50.0 μL cuvette having a 1.00 cm pathlength. Figure S6. Effect of Cl^−^ on the stability of AgNDs. (a) UV-vis spectra of the as-prepared AgND-Ab dispersed in PBS (with Cl^−^ present) and measured after 1 h and 1 day. (b) UV-vis spectra of as-prepared AgND-Ab dispersed in PB (no Cl^−^) and measured after 1 and 2 weeks. The AgNDs were stored at 4 °C between measurements. All spectra were collected using a 50.0 μL cuvette having a 1.00 cm pathlength. Figure S7. Representative dark-field STEM image of the nanorings produced after Galvanic Exchange.
