# Supplementary material for: Paper Biosensor for the Detection of NT-proBNP Using Silver Nanodisks as Electrochemical Labels
Source: Nanomaterials (Basel). 2022 Jun 30;12(13):2254. doi: 10.3390/nano12132254 (PMC9268099; doi:10.3390/nano12132254)
Supplement: Supplementary file 1 [file nanomaterials-12-02254-s001.zip › nanomaterials-1746396-supplementary.pdf]

nanomaterials

Supplementary Materials

Paper Biosensor for the Detection of NT-proBNP Using Silver Nanodisks as Electrochemical Labels

Yi Peng, Nikhil Raj, Juliette W. Strasser and Richard M. Crooks *

Department of Chemistry, The University of Texas at Austin, 100 E. 24th St., Stop A1590, Austin, TX 78712-1224, USA; peng.yi@wayne.edu (Y.P.); nkrjnishu01@gmail.com (N.R.); jstrasser@utexas.edu (J.W.S.)

* Correspondence: crooks@cm.utexas.edu

TABLE OF CONTENTS

Page	Description
S2	Additional experimental information.
S2	TEM image of the Ag seeds used to prepare the AgNDs (Figure S1).
S3	Optimization of Ag electrodeposition time and number of GE cycles for the MμB-AgND MC assay (Figure S2)
S3	HRTEM and the corresponding SAED characterization of the AgND edge. (Figure S3)
S3	Calibration curve of UV-vis absorption vs. AgND concentration. (Figure S4)
S4	Effect of PVP on the AgND stability (Figure S5)
S4	Effect of Cl^- on the stability of AgNDs (Figure S6)
S5	Dark field STEM image of the nanorings produced after GE (Figure S7)
S5	Reference

Electrode fabrication. A slight modification of a previously published procedure was used to fabricate the electrodes [1]. Specifically, they were fabricated by stencil printing carbon paste onto wax-patterned sheets of chromatography paper that had been printed using a Xerox ColorQube 8570DN printer. Following printing, the wax was melted through the thickness of the paper by placing it in an oven at 120 °C for 25.0 s. Photopaper was glued to the back of the wax-printed chromatography paper to improve rigidity, and then it was cut into 12 rectangles (2.0 cm × 5.0 cm, each). A stencil for defining the 3.0 mm-diameter disk-shaped working electrode, hook-shaped carbon quasi-reference electrode, and counter electrode was created using CorelDRAW (Ottawa, ON, Canada). The stencil was cut into a thin plastic sheet of transparency film using an Epilog laser engraving system (Zing 16). Finally, the stencil was placed over the paper (wax side up), the electrodes were printed through the stencil using the conductive carbon paste, and then they were left to dry in air for 14 h.

The working electrode was further modified by drop-casting 11 ± 2 nm diameter spherical AuNPs (sAuNPs) onto the working electrode using the protocol described in previously published paper [1]. Specifically, 10.0 μL of a diluted sAuNP solution was placed onto the working electrode such that a total of ~1.0×10^{10} sAuNPs were deposited. The sAuNP stock solution had an atomic Au concentration of ~0.87 mM or ~1.0×10^{13} sAuNPs/mL).

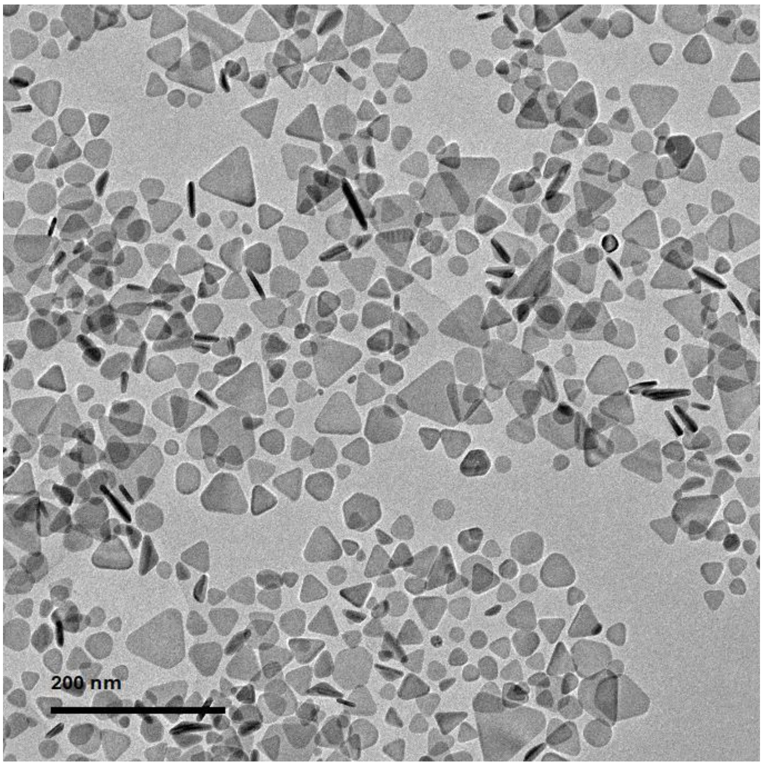

Figure S1. Representative TEM image of the Ag seeds used to prepare the AgNDs.

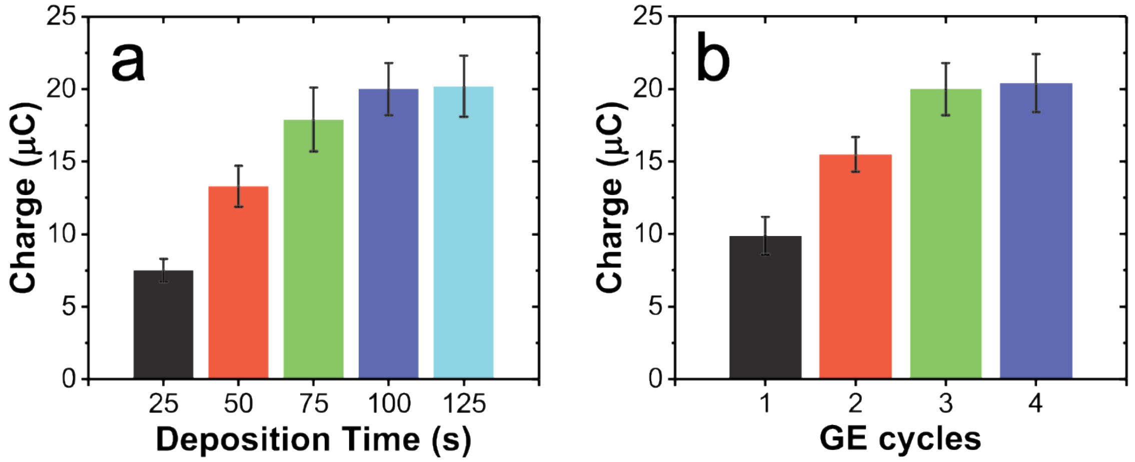

Figure S2. Optimization of the Ag electrodeposition time and number of GE cycles for the MμB-AgND MC assay. The experiment was carried out using 10 pM AgNDs and different (**a**) electrodeposition times and (**b**) number of GE cycles prior to the ASV measurements.

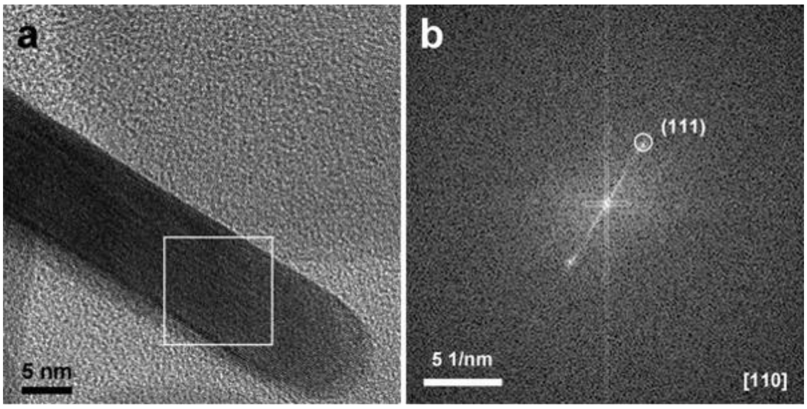

Figure S3. (**a**) HRTEM image of part of the AgND narrow edge. (**b**) The corresponding selected area electron diffraction (SAED) pattern via fast Fourier transform of the particle shown in (**a**).

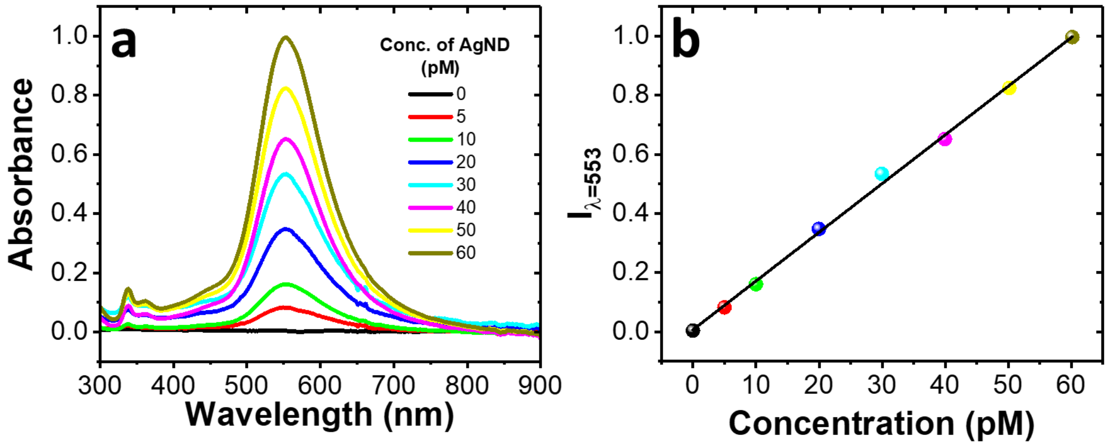

Figure S4. Calibration curve established using the absorption intensity of the major peak at 553 nm and the concentration of AgNDs obtained from ICP-MS. (**a**) The UV-vis spectra of AgND solutions having the concentrations indicated in the legend. All spectra were collected using a 50.0 μL cuvette having a 1.00 cm pathlength. (**b**) The corresponding linear plot with a linear function of $y = 0.0165x + 0.0071$, where y is the absorption intensity and x is the concentration of AgND with a unit of pM.

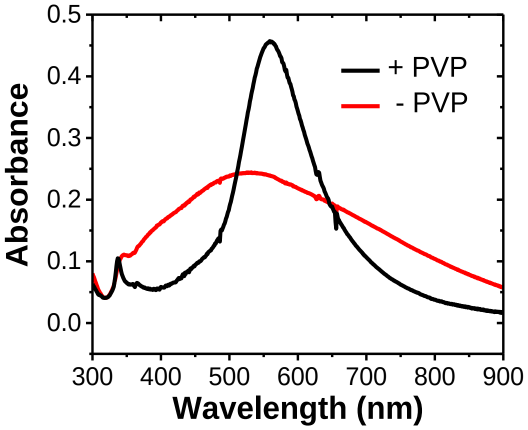

Figure S5. Effect of PVP on AgND stability. The UV-vis spectra of the AgND-Ab conjugate (~27 pM) in the presence (black curve) and absence (red curve) of PVP. The spectra were collected in phosphate buffer solutions (no Cl^- present). All spectra were collected using a 50.0 μL cuvette having a 1.00 cm pathlength.

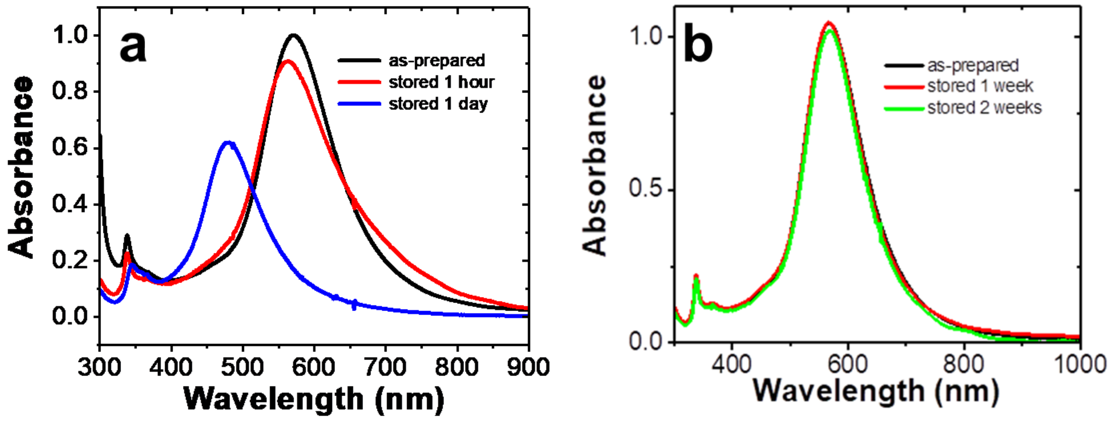

Figure S6. Effect of Cl^- on the stability of AgNDs. (**a**) UV-vis spectra of the as-prepared AgND-Ab dispersed in PBS (with Cl^- present) and measured after 1 h and 1 day. (**b**) UV-vis spectra of as-prepared AgND-Ab dispersed in PB (no Cl^-) and measured after 1 and 2 weeks. The AgNDs were stored at 4 °C between measurements. All spectra were collected using a 50.0 μL cuvette having a 1.00 cm pathlength.

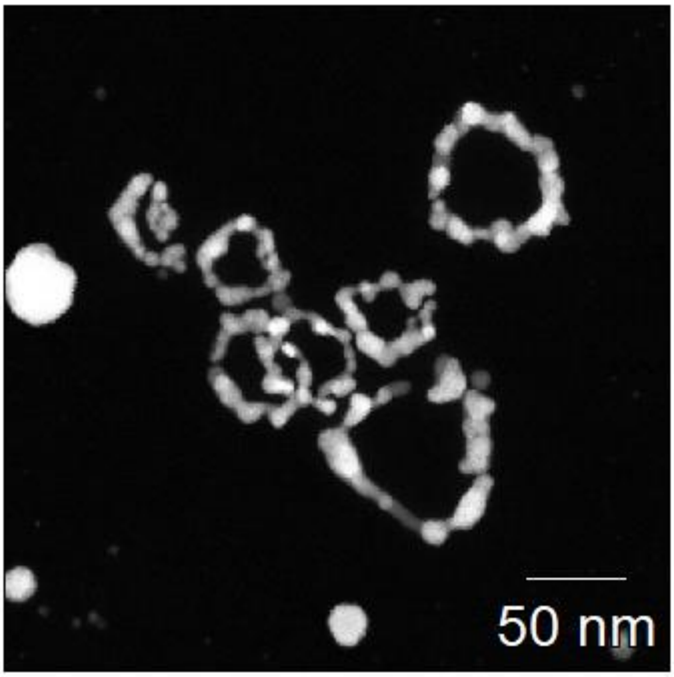

Figure S7. Representative dark-field STEM image of the nanorings produced after GE.

Reference

1. Peng, Y.; Rabin, C.; Walgama, C.T.; Pollok, N.E.; Smith, L.; Richards, I.; Crooks, R.M. Silver Nanocubes as Electrochemical Labels for Bioassays. *ACS Sens.* **2021**, *6*, 1111–1119.
